# α-Taxilin Interacts with Sorting Nexin 4 and Participates in the Recycling Pathway of Transferrin Receptor

**DOI:** 10.1371/journal.pone.0093509

**Published:** 2014-04-01

**Authors:** Hiroshi Sakane, Yukimi Horii, Satoru Nogami, Yoji Kawano, Takako Kaneko-Kawano, Hiromichi Shirataki

**Affiliations:** Department of Molecular and Cell Biology, Graduate school of Medicine, Dokkyo Medical University, Mibu, Tochigi, Japan; Thomas Jefferson University, United States of America

## Abstract

Membrane traffic plays a crucial role in delivering proteins and lipids to their intracellular destinations. We previously identified α-taxilin as a binding partner of the syntaxin family, which is involved in intracellular vesicle traffic. α-Taxilin is overexpressed in tumor tissues and interacts with polymerized tubulin, but the precise function of α-taxilin remains unclear. Receptor proteins on the plasma membrane are internalized, delivered to early endosomes and then either sorted to the lysosome for degradation or recycled back to the plasma membrane. In this study, we found that knockdown of α-taxilin induced the lysosomal degradation of transferrin receptor (TfnR), a well-known receptor which is generally recycled back to the plasma membrane after internalization, and impeded the recycling of transferrin. α-Taxilin was immunoprecipitated with sorting nexin 4 (SNX4), which is involved in the recycling of TfnR. Furthermore, knockdown of α-taxilin decreased the number and length of SNX4-positive tubular structures. We report for the first time that α-taxilin interacts with SNX4 and plays a role in the recycling pathway of TfnR.

## Introduction

Membrane traffic is essential for delivering proteins and lipids to their intracellular destinations and regulates diverse signal transduction pathways. To transport molecules to their destinations, transport vesicles are generated from the donor membrane and subsequently fuse with the target membrane [1]–[3]. We previously identified α-taxilin as a binding protein of the syntaxin family, which is involved in intracellular vesicle traffic [4]. α-Taxilin is a member of the taxilin family, which is composed of at least three proteins, α-, β- and γ-taxilin [5], and the structure of taxilin family proteins is characterized by a long coiled-coil domain. Previous studies have revealed that α- and γ-taxilin are ubiquitously expressed [4], [5], while β-taxilin, which was originally identified as a MDP77, is expressed in skeletal muscle and heart [6], [7]. It was reported that α-taxilin is overexpressed in hepatocellular carcinoma, renal cell carcinoma and glioblastoma [8]–[10]. Furthermore, we recently reported that α-taxilin binds to polymerized tubulin and partly colocalizes with α-tubulin [11], but the precise function of α-taxilin remains to be elucidated.

Receptor proteins on the plasma membrane are internalized and delivered to early endosomes, and then the internalized receptors are sorted to the lysosome for degradation or recycled back to the plasma membrane [12]. Recycling of internalized proteins is thought to be required for the regulation of plasma membrane composition and is involved in various cellular processes. Studies of the transferrin receptor (TfnR), a well-known receptor that is recycled back to the plasma membrane after internalization from early endosomes or recycling endosomes, have contributed to much of the current understanding of the recycling pathway [12], [13]. TfnR and its ligand, transferrin (Tfn), are required for the uptake of iron, which is involved in fundamental cellular processes such as oxygen transport, energy metabolism and DNA synthesis [14]. Therefore, the regulation of TfnR and Tfn recycling is thought to be important for the fundamental cellular processes. TfnR binds to diferric transferrin (Tfn) on the plasma membrane, and after the internalization of Tfn-TfnR complex, irons are released from Tfn in an acidic environment [13], [15]. Then, Tfn-TfnR complex is recycled back to the plasma membrane and Tfn is released from TfnR.

Members of the sorting nexin (SNX) family are characterized by the presence of phospholipid-binding phox homology domain and are involved in various aspects of intracellular vesicle traffic [16]–[19]. Previous reports have shown that some SNX proteins associate with a variety of receptors [18], [20], suggesting that SNX proteins play a role in intracellular vesicle traffic of receptors, and that sorting of proteins is regulated by members of the SNX family; knockdown of SNX4 causes the lysosomal degradation of TfnR [21], and SNX17 is required for the recycling of β1-integrin and low-density lipoprotein receptor-related protein (LRP) [22]–[24]. SNX4 is localized to tubular and vesicular structures that overlap with early endosomes and recycling endosomes, suggesting that SNX4 is involved in the sorting of TfnR from early endosomes to recycling endosomes [21], [25], [26]. It was also reported that SNX4 associates with clathrin and amphiphysin, which are molecules involved in endocytic trafficking [27], [28].

In this study, we found that knockdown of α-taxilin induced the lysosomal degradation of TfnR and impeded the recycling of Tfn. We also found that α-taxilin was immunoprecipitated with SNX4 and knockdown of α-taxilin decreased the number and length of SNX4-positive tubular structures. Our results demonstrate that α-taxilin is a binding partner of SNX4 and plays a role in the recycling pathway of TfnR and Tfn.

## Materials and Methods

### Materials and chemicals

An anti-α-taxilin antibody (200 ng/ml for immunofluorescence; 30 ng/ml for western blotting) was prepared as described previously [29]. Anti-clathrin heavy chain (610499, 1∶5000 for western blotting), anti-epidermal growth factor receptor (EGFR) (610016, 1∶1000 for western blotting) and anti-EEA1 (610456, 1∶500 for immunofluorescence) antibodies were purchased from BD Biosciences (San Jose, CA). Anti-TfnR (136800, 1∶500 for immunofluorescence; 1∶5000 for western blotting) and anti-GFP (A6455, 1∶10000 for western blotting) antibodies were purchased from Invitrogen (Carlsbad, CA). Anti-HA (MMS-101P, 1∶500 for immunofluorescence) and anti-myc (562, 1∶5000 for western blotting) antibodies were purchased from Covance (Princeton, NJ) and MBL (Nagoya, Japan), respectively. Anti-HA (561, 1∶1000 for western blotting) and anti-LRP6 antibodies (2560S, 1∶1000 for western blotting) were purchased from MBL and Cell Signaling Technology (Danvers, CA), respectively. An anti-α-tubulin antibody (ab15246, 1∶10000 for western blotting) was purchased from Abcam (Cambridge, UK). Bafilomycin A1, leupeptin and lactacystin were purchased from Wako Pure Chemicals (Osaka, Japan), Sigma-Aldrich (St. Louis, MO) and Calbiochem (San Diego, CA), respectively. Epidermal growth factor (EGF) was purchased from R&D systems (Minneapolis, MN). Wnt3a conditioned medium, a filtered medium of L cells stably expressing Wnt3a, was a kind gift from Akira Kikuchi (Osaka University, Osaka, Japan).

### cDNA constructs

Full length human *SNX1*, *SNX4* and *SNX17* cDNA fragments were amplified by PCR from HeLaS3 cDNA and introduced into the EcoRI and SalI sites of a pEGFP-C2 vector, XhoI and KpnI sites of a pEGFP-C3 vector, and EcoRI and SalI sites of a pEGFP-C2 vector, respectively. Full length *SNX4* cDNA fragment was introduced into the EcoRV and XhoI sites of a pcDNA-HA vector. cDNA fragments of *SNX4* corresponding to amino acids 1–402, 1–189, 292–450 and 403–450 were also amplified by PCR and introduced into the XhoI and KpnI sites of a pEGFP-C3 vector.

### Cell culture and transfection

HeLaS3 and HEK293 cells were grown in DMEM supplemented with 10% fetal calf serum at 37°C in a 5% CO_2_ incubator. Transfection of expression vectors into the cells was performed using Lipofectamine2000 (Invitrogen) according to the manufacturer's protocol. To knockdown α-taxilin or SNX4, HeLaS3 cells were transfected with 10 nM small interfering RNA (siRNA) using RNAi max (Invitrogen) according to the manufacturer's protocol and cultured for 48 h. Negative control, α-taxilin#2 (CCTGCTTGAGATGGCTGAGGAGAAA), α-taxilin#3 (GGAAGGAGATCACGTTGCTGATGCA), α-taxilin#4 (CCTCGCACTTCCAGGTGACACTGAA), SNX4#1 (GAATTCAAGTTTGGACCAATGCTAA) and SNX4#2 (GAAACATATACTGCTTACCTCATTG) stealth siRNAs were purchased from Invitrogen. For the isolation of HeLaS3 cells stably expressing control or α-taxilin short hairpin RNA (shRNA) (AAGGAGAUCACGUUGCUGAUG), pSUPER-neo/GFP vector (Oligoengine, Seattle, WA) containing target sequence was transfected into HeLaS3 cells. Transfectants of HeLaS3 cells were selected with 0.5 mg/ml of G418 (Nacalai Tesque, Kyoto, Japan), and the colony of resistant cells was isolated.

### Western blotting

Cells were lysed in lysis buffer [1% NP-40, 20 mM Tris/HCl (pH 8.0), 137 mM NaCl and 10% Glycerol] and the protein concentration of cell lysates was determined using the DC protein assay kit (Bio-Rad, Hercules, CA). Cell lysates were prepared for SDS-PAGE analysis and after western blotting, immunoreactive bands were detected using Clarity Western ECL Substrate (Bio-Rad).

### Immunoprecipitation

Transfected HEK293 cells were lysed in 1 ml of the lysis buffer, and then 450 μl of cell lysates were immunoprecipitated with 1 μg of a nonimmune IgG, an anti-myc or an anti-HA antibody using protein G sepharose (GE healthcare, Little Chalfont, Buckinghamshire, UK) at 4°C for 2 h. The immunoprecipitates were washed three times with the lysis buffer and used for western blot analysis. The results shown are representative of three independent experiments.

### Internalization assay using cleavable sulfo-NHS-SS-biotin

Cell surface proteins of HeLaS3 cells were biotinylated with 0.5 mg/ml sulfo-NHS-SS-biotin (Thermo scientific, Rockford, IL) in PBS containing 1 mM MgCl_2_ and 1 mM CaCl_2_ at 4°C for 30 min, and excess biotin was quenched with PBS containing 1 mM MgCl_2_, 1 mM CaCl_2_ and 50 mM NH_4_Cl. Then, the cells were incubated at 37°C in DMEM for various time periods. The remaining biotin on the cell surface was stripped with 50 mM sodium 2-mercaptoethanesulfonate (MesNa) (Sigma-Aldrich) in 100 mM Tris-HCl (pH 8.6) containing 100 mM NaCl and 2.5 mM CaCl_2_ at 4°C for 30 min. The cells were lysed in 0.5 ml of TNE buffer [25 mM Tris-HCl (pH 7.5), 150 mM NaCl and 5 mM EDTA] containing 1% Triton X-100 and 0.4% sodium deoxycholate, and biotinylated proteins were precipitated with neutravidin-agarose beads (Thermo Scientific) at 4°C for 2 h. The precipitates were prepared for western blot analysis.

### Biotinylation recycling assay using cleavable sulfo-NHS-SS-biotin

Cell surface proteins of HeLaS3 cells were biotinylated with 0.5 mg/ml sulfo-NHS-SS-biotin as described above. The cells were stimulated with Wnt3a conditioned medium at 37°C for 30 min to induce the internalization of LRP6. After then, the remaining biotin on the cell surface was stripped with 50 mM MesNa in 100 mM Tris-HCl (pH 8.6) containing 100 mM NaCl and 2.5 mM CaCl_2_ at 4°C for 30 min. The cells were further incubated without Wnt3a at 37°C for 30 min to allow internalized proteins to recycle back to the cell surface. Biotinylated proteins recycled back to the cell surface were again stripped of biotin using 50 mM MesNa. The cells were lysed in 0.5 ml of TNE buffer containing 1% Triton X-100 and 0.4% sodium deoxycholate, and biotinylated proteins were precipitated with neutravidin-agarose beads at 4°C for 2 h. The precipitates were prepared for western blot analysis.

### Tfn recycling assay

HeLaS3 cells were serum starved for 3 h in DMEM containing 0.2% BSA, and then the cells were incubated with 15 μg/ml Alexa Fluor 488-conjugated Tfn (Tfn-488) (Invitrogen) at 37°C for 1 h. In the case of treatment with leupeptin and lactacystin, leupeptin (200 μg/ml) and lactacyastin (10 μM), respectively, were added in the medium 1 h prior to Tfn-488 labeling. After unbound Tfn-488 was washed out, the cells were incubated at 37°C for various time periods in normal DMEM in the presence or absence of each reagent to observe the recycling of Tfn-488 and processed for immunocytochemistry. The signal intensity of Tfn-488 per μm^2^ in at least 20 cells was measured using FV10-ASW 3.0 software (Olympus, Tokyo, Japan) from three independent experiments.

### Immunocytochemistry

Cells grown on coverslips were fixed in PBS containing 3.7% formaldehyde, and then cells were permeabilized with PBS containing 0.1% (w/v) Triton X-100, 0.2% BSA and 0.05% NaN_3_. Cells were treated with the indicated antibodies for 1 h. After washing three times with PBS, cells were treated with the secondary antibody for 1 h. To immunostain HA-SNX4 in HeLaS3 cells, Can Get Signal (Toyobo, Osaka, Japan) was used to dilute the antibody. Cells were observed using an FV10i confocal laser-scanning fluorescent microscope (Olympus). The number and length of SNX4-positive tubular structures in the section of cells were measured using FV10-ASW 3.0 software. HA-SNX4 is localized to tubular and punctate structures, and the diameter of HA-SNX4-positive punctate structure is less than 1 μm. Therefore, we defined HA-SNX4-positive elongated structure longer than 2 μm as a HA-SNX4-positive tubular structure.

### Reverse transcription (RT)-PCR

Total RNA was isolated from HeLaS3 cells using NucleoSpin RNA II (TaKaRa, Shiga, Japan). The RNA sample (2 μg) was reverse transcribed using MuLV reverse transcriptase (Applied Biosystems, Foster City, CA) in a total volume of 20 μl. Quantitative RT-PCR was performed using light cycler nano (Roche Diagnostics, Basal, Switzerland). Aliquots (0.25 μl) of the reverse transcription products were amplified in a reaction mixture (20 μl) containing FastStart Essential DNA Green Master (Roche Diagnostics) and 0.5 μM primer. Forward and reverse primers were as follows: human *TFNR*, TGAAGAGAAAGTTGTCGGAGAAA and CAGCCTCACGAGGGACATA; human *GAPDH*, CCTGTTCGACAGTCAGCCG and CGACCAAATCCGTTGACTCC; human *SNX4*, CTGGAAGGAGACTGTGAATGAA and TTGCATTAAGCGCTTTTAACC.

### Subcellular fractionation

Subcellular fractionation was performed as described previously [11]. Briefly, HeLaS3 cells transfected with control or SNX4 siRNA were suspended in buffer A [10 mM Tris/HCl (pH 7.5), 1 mM EDTA and 0.25 M sucrose], and homogenized with 15 strokes in a ball homogenizer (clearance: 0.012 mm). The homogenate was centrifuged at 1,000 *g* for 10 min at 4°C and the supernatant was used as the post-nuclear supernatant. The post-nuclear supernatant was further centrifuged at 100,000 *g* for 1 h at 4°C to separate the cytosol fraction (supernatant) and membrane fraction (pellet). The membrane fraction was resuspended in a comparable amount of buffer A, and the cytosol and membrane fractions were prepared for western blot analysis.

### Statistical analysis

Statistical analyses were carried out using Student's *t*-test. A *P*-value of less than 0.05 was considered statistically significant.

## Results

### Knockdown of α-taxilin decreases the protein level of TfnR

**Figure 1:**
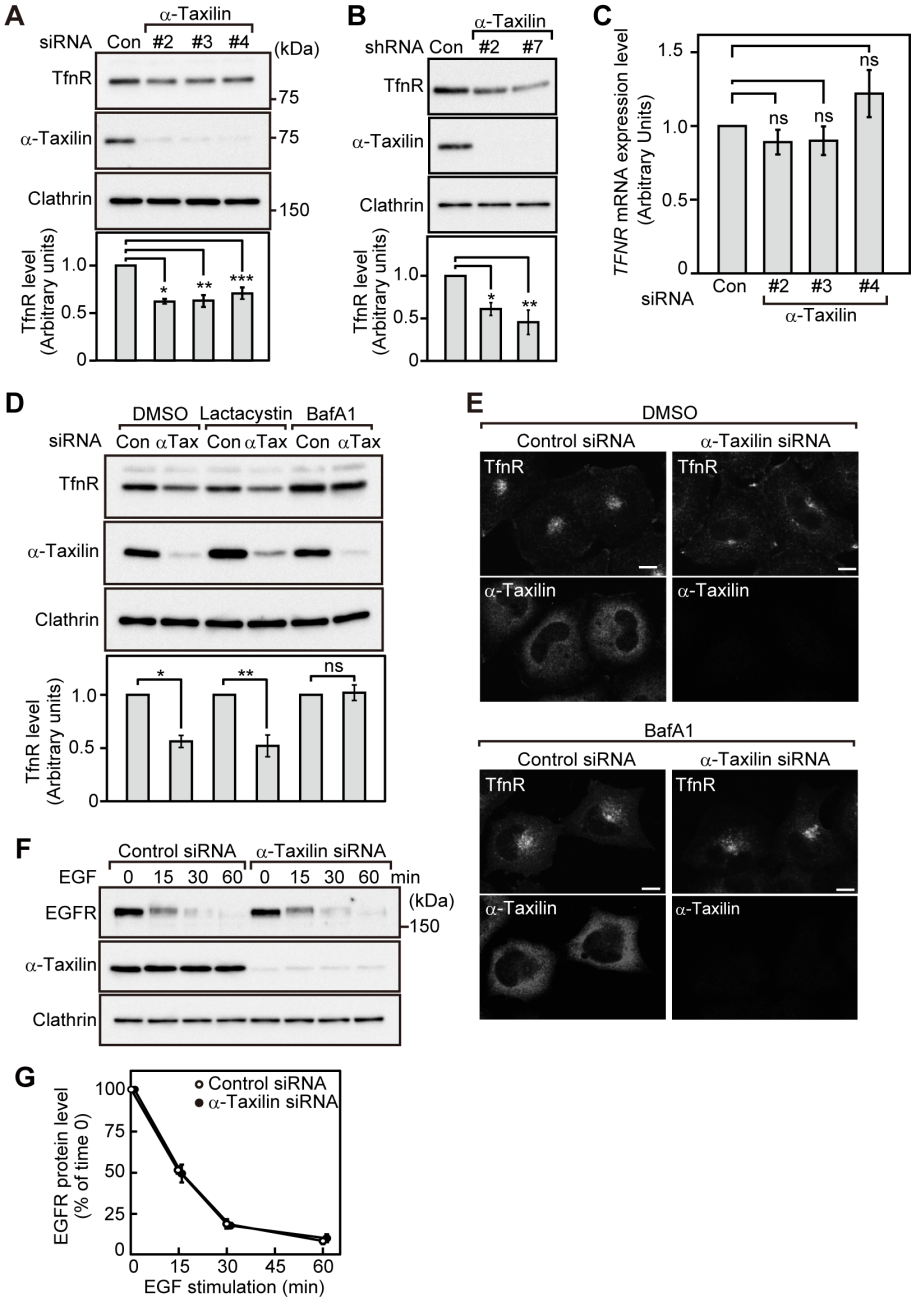
Knockdown of α-taxilin induces the degradation of TfnR. (A) Top: HeLaS3 cells transfected with control (Con) or α-taxilin siRNA (#2, #3 and #4) were lysed, and the cell lysates were probed with anti-TfnR, anti-α-taxilin and anti-clathrin heavy chain antibodies. The results shown are representative of three independent experiments. Bottom: the amount of TfnR was quantified using Image J software. The results shown are means ± s.e.m. of the ratio of TfnR in α-taxilin knockdown cells to TfnR in control cells from three independent experiments. *, P<0.0005; **, P<0.005; ***, P<0.005, by Student's *t*-test. (B) Top: HeLaS3 cells stably expressing control (Con) or α-taxilin shRNA (#2, #7) were lysed, and the cell lysates were probed with the indicated antibodies. The results shown are representative of three independent experiments. Bottom: the amount of TfnR was quantified using Image J software. The results shown are means ± s.e.m. of the ratio of TfnR in α-taxilin knockdown cells to TfnR in control cells from three independent experiments. *, P<0.005; **, P<0.005, by Student's *t*-test. (C) Total RNA was extracted from HeLaS3 cells transfected with control (Con) or α-taxilin siRNA (#2, #3 and #4) for 48 h, and *TFNR* and *GAPDH* mRNA were analyzed by RT-PCR. The ratio of the *TFNR* mRNA level relative to the *GAPDH* mRNA level was expressed as arbitrary units. *TFNR* mRNA level relative to *GAPDH* mRNA level in control HeLaS3 cells was set to 1.0. The results shown are means ± s.e.m. from three independent experiments. Ns, not significant, by Student's *t*-test. (D) Top: HeLaS3 cells transfected with control (Con) or α-taxilin (αTax) siRNA (#3) were treated with 0.1% DMSO, 10 μM lactacystin or 100 nM bafilomycin A1 (BafA1) for 24 h. The cell lysates were probed with the indicated antibodies. The results shown are representative of three independent experiments. Bottom: the amount of TfnR was quantified using Image J software. The results shown are means ± s.e.m. of the ratio of TfnR in α-taxilin knockdown cells to TfnR in control cells from three independent experiments. *, P<0.005; **, P<0.01; ns, not significant, by Student's *t*-test. (E) HeLaS3 cells transfected with control or α-taxilin siRNA (#3) were treated with 0.1% DMSO or 100 nM bafilomycin A1 (BafA1) for 24 h, and then the cells were immunostained with anti-TfnR and anti-α-taxilin antibodies. The results shown are representative of three independent experiments. Scale bars, 10 μm. (F) HeLaS3 cells transfected with control or α-taxilin siRNA (#3) were serum starved for 3 h, and then the cells were stimulated with EGF (100 ng/ml) for the indicated time periods. Cell lysates were probed with the indicated antibodies. The results shown are representative of three independent experiments. (G) The amount of EGFR in (F) was quantified using Image J software. The results shown are means ± s.e.m. of the ratio of EGFR at each time point to EGFR at time zero from three independent experiments. Values at time zero are set to 100%. *P*-values (control cells vs. α-taxilin knockdown cells at 15, 30, 60 min) determined by Student's *t*-test was not significant.

To understand the function of α-taxilin in receptor sorting, we depleted α-taxilin from HeLaS3 cells using siRNA and observed the protein level of TfnR, which is generally recycled back to the plasma membrane after internalization. Knockdown of α-taxilin in HeLaS3 cells decreased the protein level of TfnR (Figure 1A). HeLaS3 cells stably expressing α-taxilin shRNA also showed a lower level of TfnR protein than control HeLaS3 cells (Figure 1B), suggesting that the decrease in TfnR protein levels is not due to the off-target effect of siRNA treatment. Furthermore, mRNA of TfnR was not decreased in HeLaS3 cells transfected with α-taxilin siRNA (Figure 1C), indicating that α-taxilin posttranscriptionally regulates the level of TfnR protein. To examine whether knockdown of α-taxilin induces the lysosomal degradation or proteasomal degradation of TfnR, we treated HeLaS3 cells with lysosome inhibitor bafilomycin A1 or proteasome inhibitor lactacystin. Knockdown of α-taxilin decreased the TfnR protein level in HeLaS3 cells treated with lactacystin, but TfnR was not decreased in the presence of bafilomycin A1 (Figure 1D). Consistent with these results, the intensity of immunofluorescence of TfnR was decreased in HeLaS3 cells transfected with α-taxilin siRNA (Figure 1E), and was recovered in the presence of bafilomycin A1 (Figure 1E). These results demonstrate that TfnR is not targeted to the proteasome, but is instead sorted to the lysosome in α-taxilin knockdown cells.

To address whether knockdown of α-taxilin generally affects the lysosomal degradation pathway, we examined the effect of α-taxilin knockdown on the EGF-induced lysosomal degradation of EGFR in HeLaS3 cells. Knockdown of α-taxilin did not inhibit or promote the degradation of EGFR in HeLaS3 cells stimulated with EGF (Figure 1F and G), suggesting that α-taxilin is not involved in the EGF-induced degradation of EGFR.

### Recycling of Tfn is impaired in α-taxilin knockdown cells

**Figure 2:**
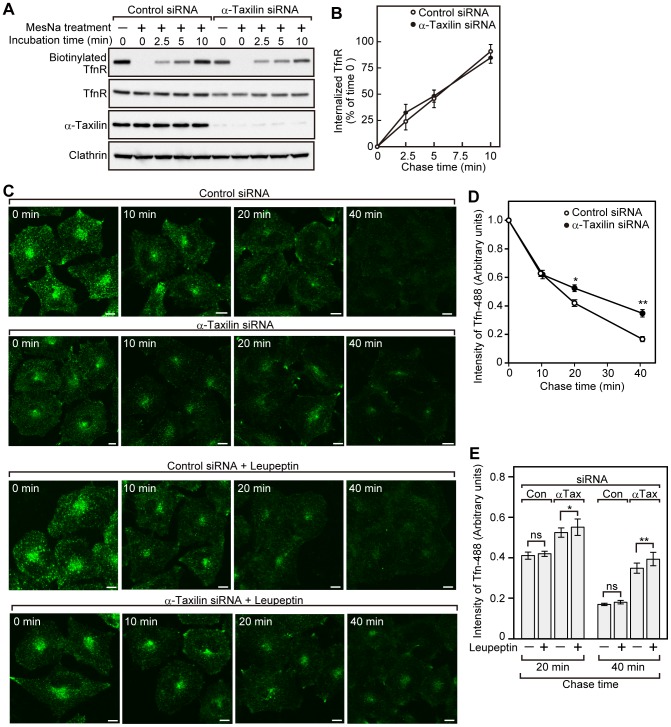
Knockdown of α-taxilin impedes the recycling of Tfn. (A) HeLaS3 cells transfected with control or α-taxilin siRNA (#3) were treated with sulfo-NHS-SS-biotin at 4°C, and then the cells were incubated at 37°C for the indicated periods of time. Cells were treated with MesNa to remove biotin remaining on the plasma membrane, and then the cell lysates were precipitated with neutravidin-agarose beads. The precipitates were probed with an anti-TfnR antibody (biotinylated TfnR). The cell lysates used for precipitation were probed with anti-TfnR, anti-α-taxilin and anti-clathrin heavy chain antibodies. The results shown are representative of three independent experiments. (B) The amount of internalized TfnR in (A) was quantified using Image J software. The results shown are means ± s.e.m. of the ratio of internalized TfnR at the indicated time periods to biotinylated TfnR at time zero without MesNa treatment from three independent experiments. *P*-values (control cells vs. α-taxilin knockdown cells at 2.5, 5, 10 min) determined by Student's *t*-test was not significant. (C) HeLaS3 cells transfected with control or α-taxilin siRNA (#3) were serum starved for 3 h, and then the cells were incubated with Tfn-488 at 37°C for 1 h. In the case of treatment with leupeptin, the cells were preincubated with leupeptin (200 μg/ml) 1 h prior to Tfn-488 labeling. After washing out unbound Tfn-488, the cells were incubated at 37°C for various time periods in the presence or absence of leupeptin (200 μg/ml). Scale bars, 10 μm. (D) The intensity of Tfn-488 signal of HeLaS3 cells untreated with leupeptin in (C) was expressed as signal intensity per unit area. At each time point, signal intensity of at least 20 cells was measured from three independent experiments. The results shown are means ± s.e.m. of the ratio of Tfn-488 at each time point to Tfn-488 at time zero. Values at time zero are set to 1.0. *P*-values (control cells vs. α-taxilin knockdown cells at 10, 20, 40 min) are determined by Student's *t*-test. *, P<0.005; **, P<0.001. *P*-values at 10 min was not significant. (E) The intensity of Tfn-488 signal of HeLaS3 cells treated or untreated with 200 μg/ml leupeptin in (C) was calculated as signal intensity per unit area. At each time point, signal intensity of at least 20 cells was measured from three independent experiments. The results shown are means ± s.e.m. of the ratio of Tfn-488 at 20 and 40 min to Tfn-488 at time zero. Values at time zero are set to 1.0. *P*-values (the cells untreated with leupeptin vs. the cells treated with leupeptin at 20 and 40 min) are determined by Student's *t*-test. *, P<0.05; **, P<0.05; ns, not significant. Con, control; α-Tax, α-taxilin.

To further confirm the effect of α-taxilin knockdown on the intracellular vesicle traffic of TfnR, we monitored the internalization of TfnR. Cell surface proteins were labeled with cleavable biotinylating agent, sulfo-NHS-SS-biotin at 4°C, and the cells were incubated at 37°C in DMEM for various times to allow internalization. Then, biotin remaining on the cell surface was stripped from the proteins using the reducing agent, MesNa. In HeLaS3 cells transfected with control siRNA, the amount of biotinylated TfnR after MesNa treatment was increased in a time-dependent manner, indicating that biotinylated TfnR was internalized in a time-dependent manner and protected from MesNa (Figure 2A and B). In HeLaS3 cells transfected with α-taxilin siRNA, the amount of biotinylated TfnR at time zero without MesNa treatment was lower than control HeLaS3 cells (Figure 2A). This is consistent with our present finding that knockdown of α-taxilin decreases the TfnR protein level. Biotinylated TfnR was internalized in a time-dependent manner in α-taxilin knockdown HeLaS3 cells as efficiently as control HeLaS3 cells (Figure 2A and B), suggesting that the internalization of TfnR is not inhibited by knockdown of α-taxilin.

To examine the effect of α-taxilin knockdown on Tfn recycling, HeLaS3 cells transfected with control or α-taxilin siRNA were incubated with Tfn-488 at 37°C for 1 h. After washing out unbound Tfn-488, the cells were incubated at 37°C for various time periods to monitor the recycling of Tfn-488. The intensity of Tfn-488 in α-taxilin knockdown cells at time zero was lower than in control cells (Figure 2C), suggesting that the decreased level of TfnR protein affected the intensity of Tfn-488 detected in the cell. Because the intensity of Tfn-488 detected in the cell was not equal between control and α-taxilin knockdown cells at time zero, Tfn-488 signal intensity at time zero was individually set to 1.0 in control and α-taxilin knockdown cells, and the intensity of Tfn-488 at the indicated time relative to that at time zero was shown in Figure 2D. In control cells, Tfn-488 signal was decreased in a time-dependent manner (Figure 2C and D). It is noted that after internalized Tfn is recycled back to the plasma membrane, it is immediately dissociated from the plasma membrane and released to the medium. However, there is a possibility that the lysosomal and proteasomal degradation pathways participate in the attenuation of Tfn-488 signal. Then, to address this issue, we examined the attenuation of Tfn-488 signal in the presence of lysosome inhibitor leupeptin or proteasome inhibitor lactacystin. Neither leupeptin treatment (Figure 2C and E) nor lactacystin treatment (Figure S1A and B) affected the attenuation of Tfn-488 signal in control cells. Therefore, these results indicate that the attenuation of Tfn-488 signal in this assay is mainly caused by the recycling of Tfn-488. In α-taxilin knockdown cells, Tfn-488 signal was also decreased in a time-dependent manner, but the decrease rate of Tfn-488 signal intensity was slower than in control cells (Figure 2C and D). As we here found that TfnR was partly sorted to the lysosome in α-taxilin knockdown cells, there is a possibility that the lysosomal degradation of Tfn-488 participates in the attenuation of Tfn-488 signal in α-taxilin knockdown cells. Really, in α-taxilin knockdown cells treated with leupeptin (Figure 2C), but not with lactacystin (Figure S1A), Tfn-488 signal was slightly increased when compared with that in untreated α-taxilin knockdown cells, suggesting that a part of internalized Tfn-488 is sorted to the lysosome and subsequently degraded in α-taxilin knockdown cells. Furthermore, the treatment with leupeptin (Figure 2E), but not with lactacystin (Figure S1B), slightly decreased the attenuation rate of Tfn-488 signal in α-taxilin knockdown cells. Taken together, these results demonstrate that Tfn-488 signal in α-taxilin knockdown cells treated with leupeptin actually show the amount of Tfn-488 that is not recycled and suggest that knockdown of α-taxilin impedes the recycling of Tfn.

To examine whether knockdown of α-taxilin affects the recycling of other receptors, we monitored the recycling of LRP6, which plays a pivotal role in Wnt β-catenin pathway [30]. It is known that LRP6 internalized with the stimulation of its ligand, Wnt3a, is recycled back to the plasma membrane [31]. We examined the effect of α-taxilin knockdown on the recycling of LRP6 in HeLaS3 cells using sulfo-NHS-SS-biotin. After biotinylation, the cells were stimulated with Wnt3a for 30 min to induce the internalization of LRP6 (Figure S2, lanes 3–5, 8–10), followed by MesNa treatment to remove biotin remaining on the cell surface (Figure S2, lanes 2–5, 7–10). In HeLaS3 cells prior to stimulation with Wnt3a, biotinylated LRP6 was detected without MesNa treatment (Figure S2, lanes 1 and 6), but was hardly detected after MesNa treatment (Figure S2, lanes 2 and 7), confirming that biotin was absolutely removed from biotinylated LRP6 on the cell surface by MesNa treatment. In HeLaS3 cells stimulated with Wnt3a, biotinylated LRP6 was internalized and the internalized biotinylated LRP6 was protected from MesNa (Figure S2, lanes 3–5, 8–10). After removing biotin from biotinylated LRP6 remaining on the cell surface by MesNa treatment, the cells were incubated for 30 min without Wnt3a to allow internalized biotinylated LRP6 to recycle back to the cell surface (Figure S2, lanes 4, 5, 9 and 10). At this time, the cell surface was again treated with MesNa to remove biotin from recycled biotinylated LRP6 (Figure S2, lanes 4 and 9) or untreated with MesNa (Figure S2, lanes 5 and 10) to monitor the degradation of internalized biotinylated LRP6. When the cells were untreated with MesNa, the amount of biotinylated LRP6 was not significantly decreased in either control or α-taxilin knockdown cell (Figure S2, lanes 5 and 10), suggesting that internalized LRP6 is hardly degraded in either cell. When the cells were treated with MesNa, biotinylated LRP6 was hardly detected in either control or α-taxilin knockdown cell (Figure S2, lanes 4 and 9), suggesting that the majority of internalized LRP6 was recycled back to the plasma membrane in both cells. These results suggest that α-taxilin is not involved in the recycling of LRP6.

### α-Taxilin interacts with SNX4

**Figure 3:**
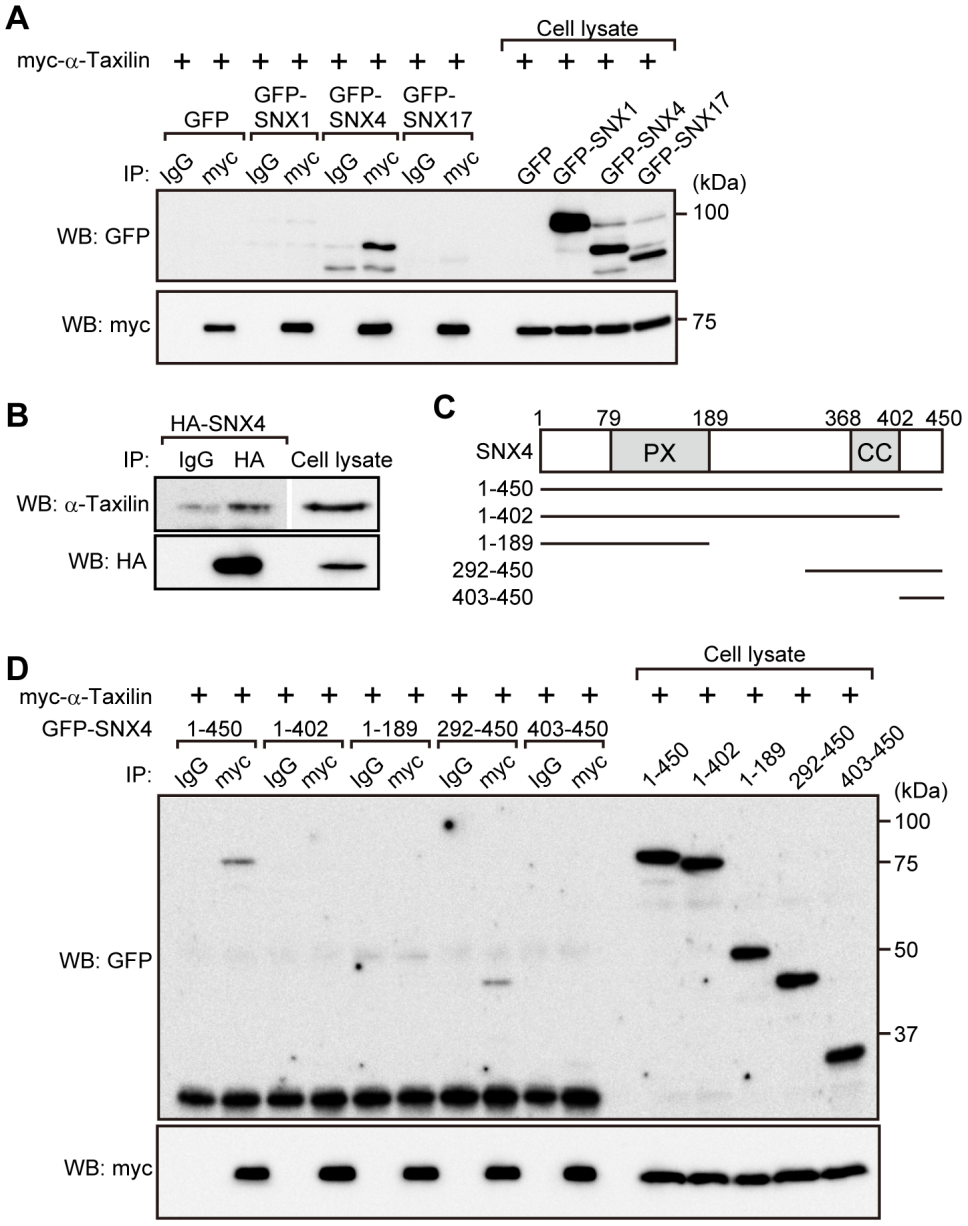
α-Taxilin immunoprecipitates with SNX4. (A) HEK293 cells expressing myc-α-taxilin and GFP-SNX1, GFP-SNX4 or GFP-SNX17 were lysed, and the cell lysates were immunoprecipitated with a nonimmune IgG or an anti-myc antibody. The immunoprecipitates and the cell lysates used for immunoprecipitation were probed with anti-myc and anti-GFP antibodies. (B) HEK293 cells expressing HA-SNX4 were lysed, and the cell lysates were immunoprecipitated with a nonimmune IgG or an anti-HA antibody. The immunoprecipitates and the cell lysates used for immunoprecipitation were probed with anti-HA and anti-α-taxilin antibodies. (C) Construction of GFP-SNX4 mutants used in (D) was shown. PX, phox homology domain; CC, coiled-coil domain. (D) HEK293 cells expressing myc-α-taxilin and GFP-SNX4 mutants indicated in (C) were lysed, and the cell lysates were immunoprecipitated with a nonimmune IgG or an anti-myc antibody. The immunoprecipitates and the cell lysates used for immunoprecipitation were probed with anti-myc and anti-GFP antibodies.

Members of the SNX family are involved in the recycling of receptors; knockdown of SNX4, but not SNX1, inhibits the recycling of TfnR [21] and SNX17 is involved in the recycling of β1-integrin and LRP [22]–[24]. To examine whether α-taxilin associates with members of the SNX family, myc-α-taxilin was expressed with GFP-SNX1, GFP-SNX4 or GFP-SNX17 in HEK293 cells and the cell lysates were immunoprecipitated with the anti-myc antibody (Figure 3A). Myc-α-taxilin was significantly immunoprecipitated with GFP-SNX4, but only slightly with GFP-SNX1 and GFP-SNX17 (Figure 3A). Furthermore, endogenous α-taxilin was also immunoprecipitated with HA-SNX4 (Figure 3B). Taken together, these results suggest that α-taxilin specifically interacts with HA-SNX4.

SNX4 is composed of an N-terminal phox homology domain and a C-terminal coiled-coil domain [17]. To determine the region of SNX4 that is required for interaction with α-taxilin, a number of SNX4 mutants fused with GFP were expressed with myc-α-taxilin (Figure 3C and D). GFP-SNX4 (residues 1–402) and GFP-SNX4 (residues 1–189) mutants failed to immunoprecipitate with myc-α-taxilin (Figure 3D). The GFP-SNX4 (403–450) mutant, which contains only the C-terminal region of SNX4 just after the coiled-coil domain, also failed to immunoprecipitate with myc-α-taxilin (Figure 3D). The GFP-SNX4 (292–450) mutant, which contains the coiled-coil domain and C-terminal region, was immunoprecipitated with myc-α-taxilin (Figure 3D). These results suggest that the C-terminal region of SNX4 containing the coiled-coil domain is required for interaction with α-taxilin.

### α-Taxilin does not localize to early endosomes and recycling endosomes

**Figure 4:**
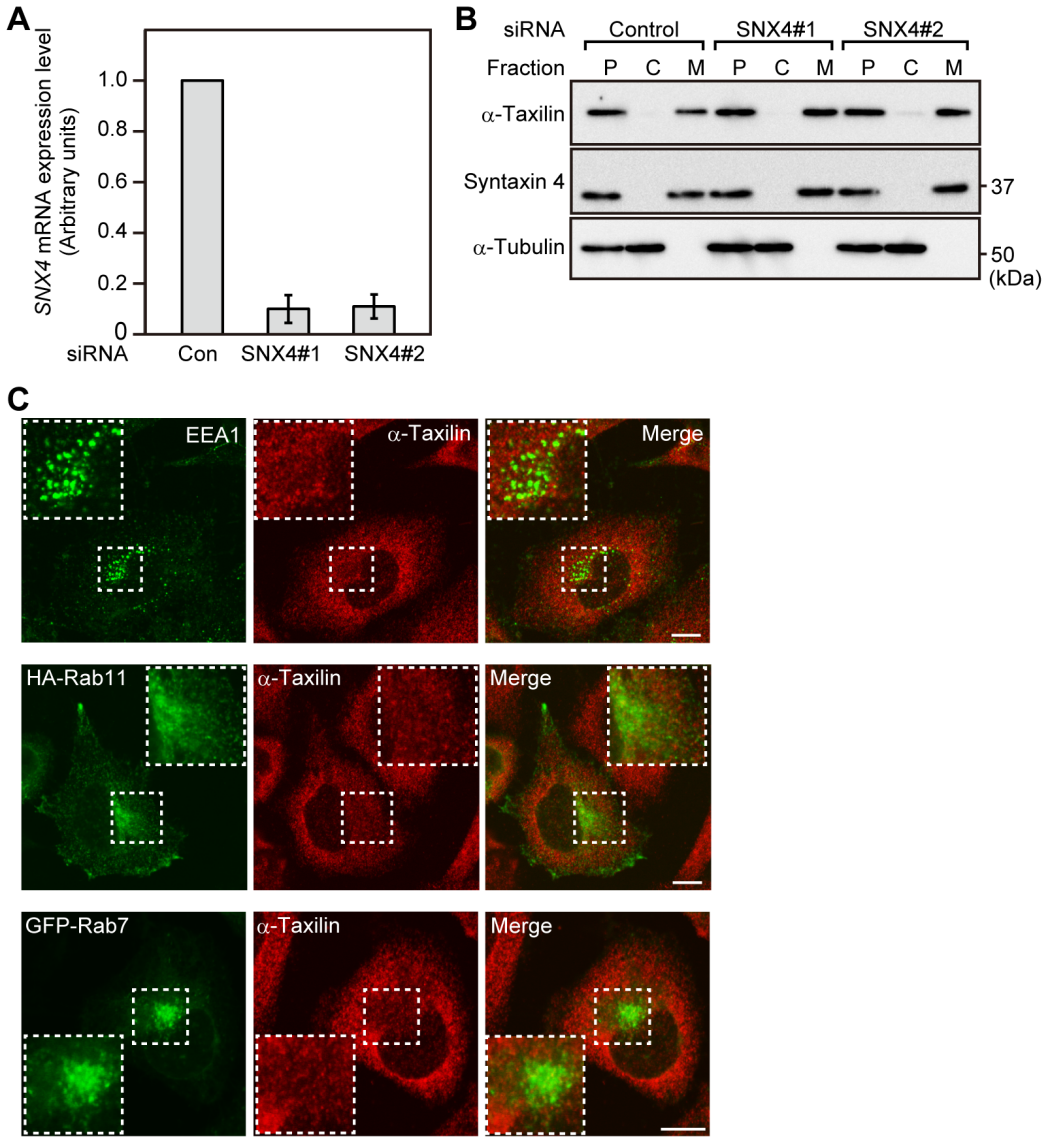
α-Taxilin does not localize to endosome structures. (A) Total RNA was extracted from HeLaS3 cells transfected with control or SNX4 siRNA (#1, #2) for 48 h, and *SNX4* and *GAPDH* mRNA were analyzed by RT-PCR. The ratio of the *SNX4* mRNA level relative to the *GAPDH* mRNA level was expressed as arbitrary units. *SNX4* mRNA level relative to *GAPDH* mRNA level in control HeLaS3 cells was set to 1.0. The results shown are means ± s.e.m. from three independent experiments. *, P<0.005; **, P<0.005, by Student's *t*-test. (B) HeLaS3 cells transfected with control or SNX4 siRNA (#1, #2) were homogenized, and the post-nuclear supernatant (P) of the homogenate was separated into the cytosol (C) and membrane (M) fractions. Fractions were probed with the indicated antibodies. Syntaxin 4 and α-tubulin were used as markers for membrane and cytosol protein, respectively. (C) Top: HeLaS3 cells were immunostained with anti-EEA1 and anti-α-taxilin antibodies. Middle: HeLaS3 cells transfected with HA-Rab11 were immunostained with anti-HA and anti-α-taxilin antibodies. Bottom: HeLaS3 cells transfected with GFP-Rab7 were immunostained with an anti-α-taxilin antibody. Boxed areas are shown at higher magnification in the inset. Scale bars, 10 μm. The results shown are representative of three independent experiments.

We previously demonstrated that α-taxilin is a peripheral membrane protein [11]. We then examined whether the membrane association of α-taxilin depends on interaction with SNX4. α-Taxilin was observed in the membrane fraction of HeLaS3 cells transfected with control or SNX4 siRNA (Figure 4A and B), suggesting that SNX4 is not required for the association of α-taxilin with membrane compartments. Because α-taxilin seems to be involved in the recycling of TfnR, we next observed whether α-taxilin localizes to early endosomes or recycling endosomes in HeLaS3 cells. α-Taxilin did not colocalize with the early endosome marker EEA1 or the recycling endosome marker HA-Rab11, and α-taxilin also did not colocalize with the late endosome marker GFP-Rab7 (Figure 4C), suggesting that α-taxilin may be not present on the membrane of early endosomes, recycling endosomes or late endosomes.

### Knockdown of α-taxilin decreases the number and length of SNX4-positive tubular structures

**Figure 5:**
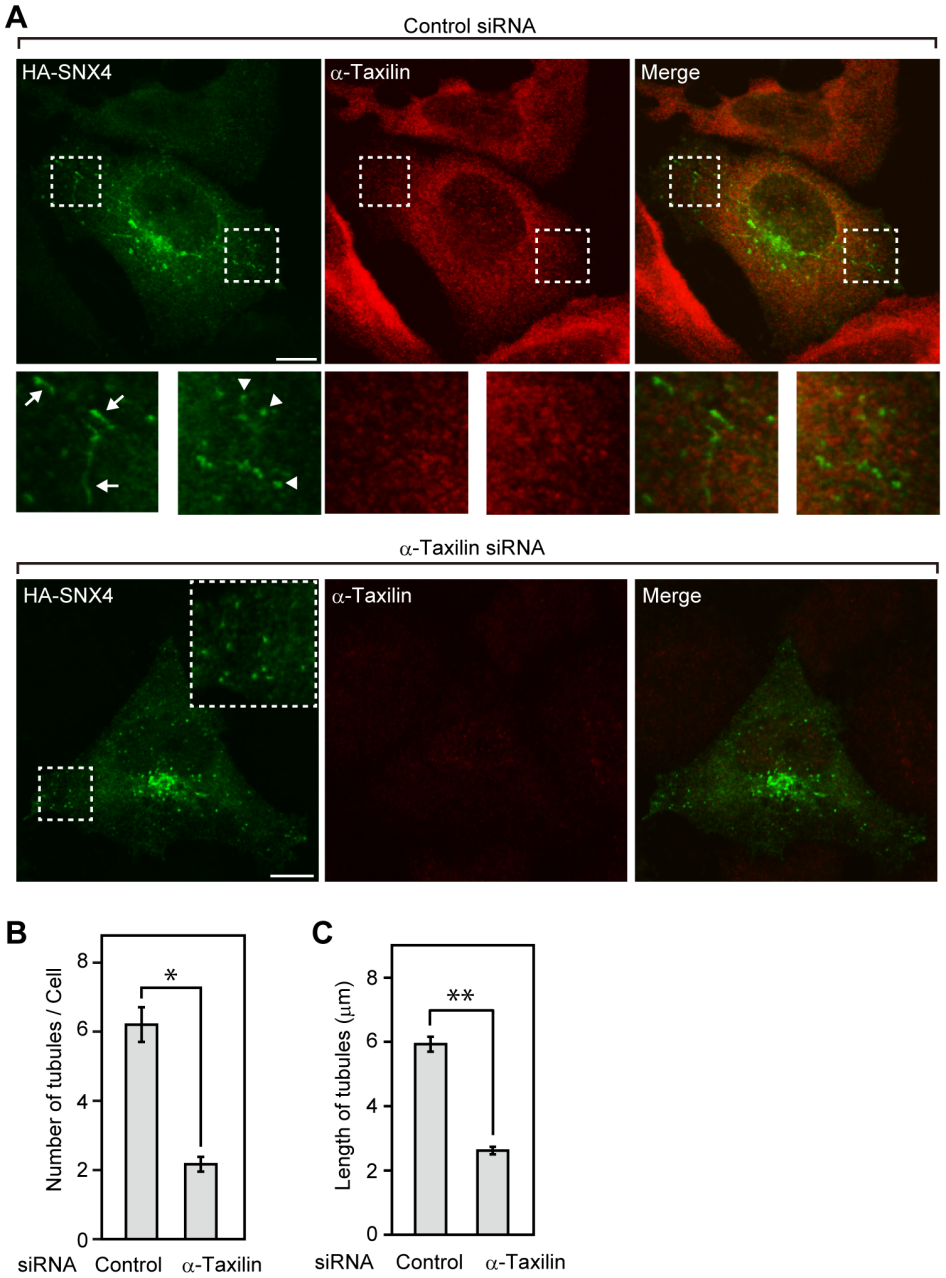
Knockdown of α-taxilin decreases the number and length of SNX4-positive tubular structures. (A) HeLaS3 cells transfected with control or α-taxilin siRNA (#3) for 24 h were further transfected with HA-SNX4, and then immunostained with anti-HA and anti-α-taxilin antibodies. In control cells, boxed areas are shown at higher magnification in the bottom. Arrows and arrowheads indicate the HA-SNX4-positive tubular structure and HA-SNX4-positive punctate structure, respectively. Scale bars, 10 μm. (B) The numbers of HA-SNX4-positive tubular structures were counted in HeLaS3 cells transfected with control or α-taxilin siRNA (#3). Control cells, 304 tubules were counted from 49 cells; α-taxilin knockdown cells, 52 tubules were counted from 24 cells. The results shown are means ± s.e.m. *, P<0.001, by Student's *t*-test. (C) The length of HA-SNX4-positive tubular structures was measured in HeLaS3 cells transfected with control or α-taxilin siRNA (#3). Control cells, the length of 304 tubules were measured from 49 cells; α-taxilin knockdown cells, the length of 52 tubules were measured from 24 cells. The results shown are means ± s.e.m. **, P<0.001, by Student's *t*-test.

Transiently expressed SNX4 localizes to punctate and tubular structures [21], [25], [26]. We examined whether knockdown of α-taxilin affect the localization of SNX4 in HeLaS3 cells. HA-SNX4 was localized to punctate and tubular structures in HeLaS3 cells transfected with control siRNA and these structures did not overlap with the distribution of α-taxilin (Figure 5A). Although HA-SNX4 was localized to punctate structures in α-taxilin knockdown cells, the number and length of HA-SNX4-positive tubular structures were decreased in HeLaS3 cells transfected with α-taxilin siRNA (Figure 5A, B and C). These results suggest that α-taxilin may be involved in the stability of HA-SNX4-positive tubular structures.

## Discussion

α-Taxilin was originally identified as a syntaxin binding protein [4], but the precise function of α-taxilin in intracellular vesicle traffic was unknown. During our investigations into the function of α-taxilin, we found that knockdown of α-taxilin in HeLaS3 cells decreased the protein level of TfnR. Knockdown of α-taxilin in COS7 cells also decreased the protein level of TfnR (data not shown), suggesting that the effect of α-taxilin depletion on the protein level of TfnR is not specific for HeLaS3 cells. TfnR protein level was not decreased when α-taxilin knockdown cells were treated with bafilomycin A1, which is generally used as a lysosome inhibitor. Depletion of α-taxilin also impeded the recycling of Tfn-488. These results demonstrate that the recycling of TfnR is impaired in α-taxilin knockdown cells and TfnR is sorted to the lysosome in the absence of α-taxilin. The results of this study demonstrate that α-taxilin is involved in the recycling pathway of TfnR.

Previous reports have shown that knockdown of SNX4 decreases the protein level of TfnR and SNX4 associates with minus end-directed microtubule motor protein Dynein through interaction with KIBRA [21]. Furthermore, transiently expressed SNX4 localizes to vesicular and tubular structures that overlap with early endosomes and recycling endosomes [21]. Because knockdown of α-taxilin or SNX4 decreased the level of TfnR protein, we hypothesized that α-taxilin is functionally related to SNX4, and then examined the interaction between α-taxilin and several members of the SNX family. α-Taxilin was immunoprecipitated with SNX4, and the C-terminal region containing the coiled-coil domain of SNX4 was required for association with α-taxilin. Although knockdown of SNX4 decreases the protein level of TfnR, SNX4 does not interact with TfnR [18], [21] and α-taxilin also did not interact with TfnR (data not shown). Therefore, it is unlikely that the function of α-taxilin is to bind to TfnR and recruit TfnR to the SNX4-positive vesicular or tubular structures. At present, it is not clear how α-taxilin regulates the proper recycling of TfnR. We previously reported that α-taxilin directly interacts with polymerized tubulin [11] and in this study we found that knockdown of α-taxilin decreases the number and length of SNX4-positive tubular structures. Therefore, it is possible that the function of α-taxilin is to connect SNX4-positive tubular structures to microtubules and support the stability of SNX4-positive tubular structures. Disruption of SNX4-positive tubular structures in α-taxilin knockdown cells may impede the proper recycling of TfnR. On the basis of the finding that knockdown of SNX4 perturbs transport of TfnR from early endosomes to recycling endosomes [21], it is suggested that SNX4-positive tubular structures are formed between early endosomes and recycling endosome. Then, it is easy to speculate that α-taxilin is present on microtubules and regulates the transport of TfnR from early endosomes to recycling endosomes. However, significant colocalization between EEA1 or HA-Rab11 and α-taxilin was not observed, suggesting that the majority of α-taxilin is not present on the membrane of early endosomes or recycling endosomes. Furthermore, the majority of α-taxilin did not colocalize with SNX4 in immunofluorescence analysis and a small portion of α-taxilin colocalized with α-tubulin [11], suggesting that the interaction between SNX4-positive tubular structures and α-taxilin, and microtubules and α-taxilin may be transient, or that only a limited region of SNX4-positive tubular structures and microtubules may be associated with α-taxilin. In either case, because α-taxilin is distributed throughout the cytoplasm, it is thought that the colocalization of α-taxilin with SNX4 is difficult to detect in our immunofluorescent analysis. Whether α-taxilin participates only in the proper recycling of TfnR or whether α-taxilin is also involved in the recycling of other proteins is unclear. However, our present finding that knockdown of α-taxilin at least did not impede the recycling of LRP6 suggests that α-taxilin is not generally involved in the regulation of receptor recycling. α-Taxilin was immunoprecipitated with SNX4, but not with SNX17, which regulates the recycling of β1-integrin and LRP. Therefore, it is likely that α-taxilin specifically interacts with SNX4, and may be involved in the proper transport of proteins that are regulated by SNX4.

Previously we found that α-taxilin is fractionated into the membrane fraction, although a predicted transmembrane region is absent in α-taxilin [11]. α-Taxilin was identified as a syntaxin binding protein, but knockdown of syntaxin 3 or syntaxin 4 in HeLa cells did not affect the membrane association of α-taxilin [11]. Knockdown of SNX4 also did not alter the membrane association of α-taxilin. Therefore, α-taxilin may interact with another membrane-associated protein and this interaction may be responsible for the membrane association of α-taxilin. At present, however, we have not identified another membrane protein that is required for the membrane association of α-taxilin and this issue will be our future work.

We observed that α-taxilin is expressed in the mouse gastrointestinal tract including the small intestine and colon [32]. mRNA analysis of SNX4 in various human tissues revealed that mRNA of SNX4 is widely expressed [18], but in the small intestine and colon where α-taxilin is expressed, the expression level of *SNX4* mRNA is low. Therefore, although α-taxilin interacts with SNX4, it is possible that α-taxilin has a function that is independent of SNX4 in these tissues, because α-taxilin also interacts with the α and β subunit of nascent polypeptide-associated complex [29], and α-taxilin was originally identified as a syntaxin binding protein [4]. Further studies are required to clarify whether α-taxilin indeed has a multifunction that depends on binding partner.

Because α-taxilin is overexpressed in hepatocellular carcinoma, renal cell carcinoma and glioblastoma [8]–[10], it is possible that overexpression of α-taxilin impairs the proper recycling of receptors. Although whether SNX4 is also overexpressed in tumor tissues where α-taxilin is overexpressed is not known, it is possible that overexpressed α-taxilin in the tumor tissues may be increase the protein level of several receptors on the plasma membrane through activation of the recycling pathway, causing the dysregulation of several signal transduction pathways. How the overexpression of α-taxilin is induced in tumor tissues and whether this overexpression is involved in the aggressiveness of the tumor remains to be elucidated. Further studies are required to clarify the role of α-taxilin in the tumor aggressiveness.

## Acknowledgments

We thank Akira Kikuchi for providing Wnt3a conditioned medium. We also thank Takuya Sasaki for donating Rab7 and Rab11 plasmids, and members of laboratories for preparing materials for experiments.

## Supporting Information

Figure S1 **Effect of lactacystin on the recycling of Tfn-488 in HeLaS3 cells.** (A) HeLaS3 cells transfected with control or α-taxilin siRNA (#3) were serum starved for 3 h, and then the cells were incubated with Tfn-488 at 37°C for 1 h. In the case of treatment with DMSO or lactacystin, the cells were preincubated with 0.1% DMSO or 10 μM lactacystin 1 h prior to Tfn-488 labeling. After washing out unbound Tfn-488, the cells were incubated at 37°C for various time periods in the presence of 0.1% DMSO or 10 μM lactacystin. Scale bars, 10 μm. (B) The intensity of Tfn-488 signal of HeLaS3 cells treated with 0.1% DMSO or 10 μM lactacystin in (A) was calculated as signal intensity per unit area. At each time point, signal intensity of at least 20 cells was measured from three independent experiments. The results shown are means ± s.e.m. of the ratio of Tfn-488 at 20 and 40 min to Tfn-488 at time zero. Values at time zero are set to 1.0. *P*-values (the cells treated with DMSO vs. the cells treated with lactacystin at 20 and 40 min) determined by Student's *t*-test was not significant.
(TIF)

Figure S2 **Knockdown of α-taxilin does not affect the recycling of LRP6.** (A) After cell-surface biotinylation using sulfo-NHS-SS-biotin (lanes 1–10), HeLaS3 cells transfected with control or α-taxilin (#3) siRNA were stimulated with Wnt3a conditioned medium for 30 min (lanes 3–5, 8–10). The remaining biotin on the cell surface was stripped using MesNa (lanes 2–5, 7–10) and then the cells were incubated without Wnt3a for 30 min (lanes 4, 5, 9 and 10). Biotinylated proteins that recycled back to the cell surface was again treated (lanes 4 and 9) or untreated (lanes 5 and 10) with MesNa. Cell lysates were precipitated with neutravidin-agarose beads. The precipitates were probed with an anti-LRP6 antibody and the cell lysates were probed with the indicated antibodies.
(TIF)
