# Supplementary figures and images for: α-Taxilin Interacts with Sorting Nexin 4 and Participates in the Recycling Pathway of Transferrin Receptor
Source: PLoS One. 2014 Apr 1;9(4):e93509. doi: 10.1371/journal.pone.0093509 (PMC3972091; doi:10.1371/journal.pone.0093509)

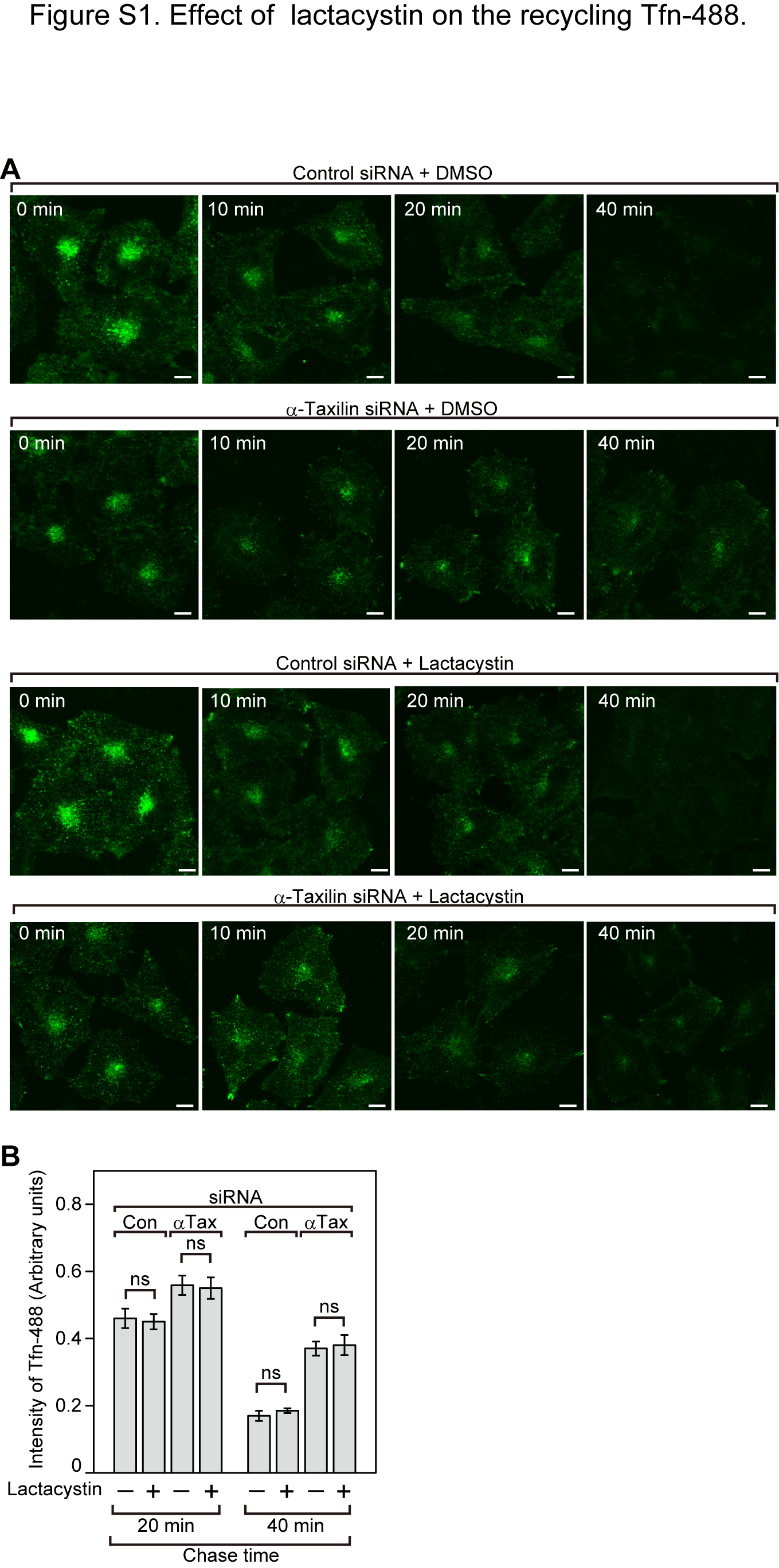

Supplement: Figure S1 — Effect of lactacystin on the recycling of Tfn-488 in HeLaS3 cells. (A) HeLaS3 cells transfected with control or α-taxilin siRNA (#3) were serum starved for 3 h, and then the cells were incubated with Tfn-488 at 37°C for 1 h. In the case of treatment with DMSO or lactacystin, the cells were preincubated with 0.1% DMSO or 10 μM lactacystin 1 h prior to Tfn-488 labeling. After washing out unbound Tfn-488, the cells were incubated at 37°C for various time periods in the presence of 0.1% DMSO or 10 μM lactacystin. Scale bars, 10 μm. (B) The intensity of Tfn-488 signal of HeLaS3 cells treated with 0.1% DMSO or 10 μM lactacystin in (A) was calculated as signal intensity per unit area. At each time point, signal intensity of at least 20 cells was measured from three independent experiments. The results shown are means ± s.e.m. of the ratio of Tfn-488 at 20 and 40 min to Tfn-488 at time zero. Values at time zero are set to 1.0. P-values (the cells treated with DMSO vs. the cells treated with lactacystin at 20 and 40 min) determined by Student's t-test was not significant. (TIF) [file pone.0093509.s001.tif]

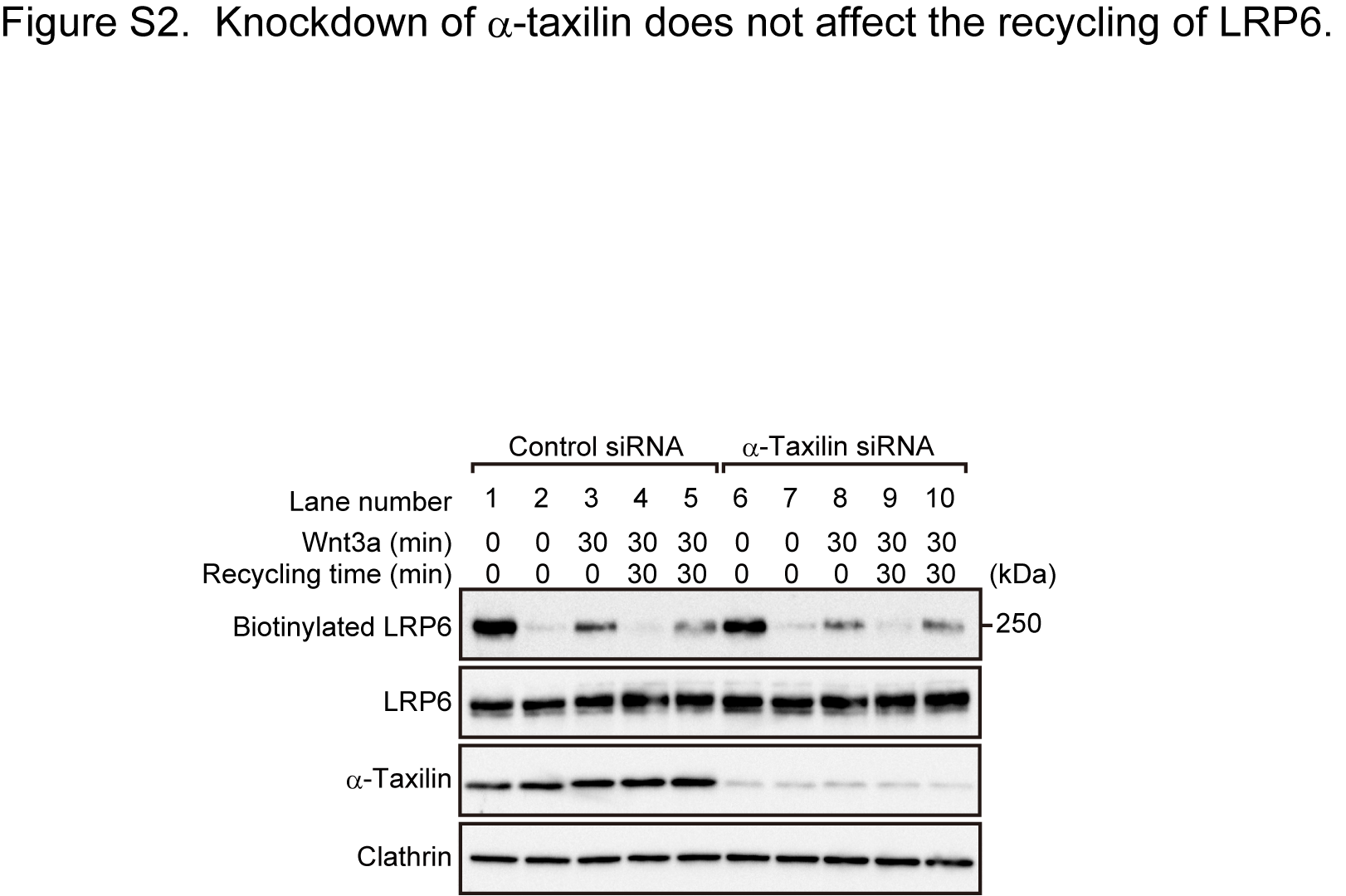

Supplement: Figure S2 — Knockdown of α-taxilin does not affect the recycling of LRP6. (A) After cell-surface biotinylation using sulfo-NHS-SS-biotin (lanes 1–10), HeLaS3 cells transfected with control or α-taxilin (#3) siRNA were stimulated with Wnt3a conditioned medium for 30 min (lanes 3–5, 8–10). The remaining biotin on the cell surface was stripped using MesNa (lanes 2–5, 7–10) and then the cells were incubated without Wnt3a for 30 min (lanes 4, 5, 9 and 10). Biotinylated proteins that recycled back to the cell surface was again treated (lanes 4 and 9) or untreated (lanes 5 and 10) with MesNa. Cell lysates were precipitated with neutravidin-agarose beads. The precipitates were probed with an anti-LRP6 antibody and the cell lysates were probed with the indicated antibodies. (TIF) [file pone.0093509.s002.tif]
